# Mechanical Performance of Advanced Composite Materials and Structures (2nd Edition)

**DOI:** 10.3390/ma19091694

**Published:** 2026-04-22

**Authors:** Yin Fan

**Affiliations:** 1 School of Aeronautics and Astronautics, Shanghai Jiao Tong University, Shanghai 200240, China; yfan1987@sjtu.edu.cn; 2 The State Key Lab of Metal Matrix Composites, Shanghai Jiao Tong University, Shanghai 200240, China

## 1. Introduction and Summary

Outstanding mechanical performance remains a central objective across a wide range of engineering fields, including aerospace [1,2,3], high-speed rail [4,5,6,7], and automotive industries [8,9,10]. With increasing economic demands and more stringent safety requirements, conventional metallic structures are progressively being replaced by advanced composite materials. However, significant challenges persist in the mechanical design of such systems, primarily due to their complex, multi-layered architectures composed of orthogonally anisotropic constituents. At present, a combination of numerical modeling, experimental characterization, and damage inspection techniques constitutes the primary framework for the design, validation, and performance assessment of composite materials and structures.

Building upon the success of the first Special Issue, “Mechanical Performance of Advanced Composite Materials and Structures”, this second edition continues to solicit high-quality contributions in this rapidly evolving field. The Guest Editor, Dr. Yin Fan, has organized this collection to encompass a broad spectrum of mechanical performance topics, including indentation, low-velocity impact, fracture, fatigue, strength, and lightning strike resistance. The targeted application domains span aerospace, wind energy, civil engineering, sensing technologies, and biomedical devices. In total, six research articles and two review papers from contributors in China, the United States, Germany, Italy, Austria, and Iran have been rigorously peer-reviewed and accepted for publication. These contributions collectively address key stages in the engineering application of composite materials, including manufacturing processes (e.g., additive manufacturing), structural design (e.g., lay-up optimization), and performance verification (e.g., numerical simulation, damage analysis, and experimental testing). The scope of the studies spans multiple length scales, ranging from nanoscale material behavior to macroscale structural performance.

## 2. Contributions

### 2.1. Indentation and Low-Velocity Impact

Indentation and low-velocity impact resistance are widely recognized as critical mechanical performance metrics for ensuring structural safety and must be carefully considered in the design of engineering structures across various application domains.

In the paper entitled “Investigating the Mechanical Behavior and Energy Absorption Characteristics of Empty and Foam-Filled Glass/Epoxy Composite Sections under Lateral Indentation”, Taghizadeh et al. [11] investigated the crashworthiness and energy absorption capabilities of composite tubes subjected to lateral indentation by steel rods aligned parallel to the specimen axis, using experimental methods. Contrary to conventional expectations, their results demonstrated that smaller-diameter foam-filled specimens exhibited superior energy absorption compared to larger ones. This behavior was primarily attributed to the more effective compression of the foam core. The study provides valuable insights into the structural response and crashworthiness of composite tubes, offering important guidance for structural design and optimization.

The primary motivation for employing composite materials in aerospace and railway vehicles lies in their potential for significant weight reduction, which directly influences fuel efficiency and operational costs. However, a major challenge limiting their broader application is their susceptibility to foreign object damage. In this context, Tian et al. [12], in their paper “Nonlinear Dynamics of Thick Hybrid Composite Laminates Subjected to Low-Velocity Impact and Various Preloading”, systematically investigated the effects of impact energy, preloading conditions, laminate thickness, and impact angle on the low-velocity impact response of preloaded hybrid composite laminates. Their study integrated numerical simulations, experimental testing, and non-destructive testing (NDT) techniques. The results revealed that, for thin laminates, the influence of preloading is primarily associated with bending deformation and failure in the back plies. In contrast, for thick laminates, preloading alters the stress state in the front plies, which consequently governs the failure behavior.

To further enhance the design flexibility and assembly efficiency of honeycomb structures, Yin et al. [13], in “Misalignment Assembly Effect on the Impact Mechanical Response of Tandem Nomex Honeycomb-Core Sandwich Structures”, examined the impact behavior of tandem honeycomb-core sandwich structures with varying misalignment assembly lengths. Unlike the findings reported by Tian et al. [12], their study included a comparative analysis between honeycomb-core sandwich structures and conventional composite laminates, demonstrating that honeycomb cores can significantly improve impact resistance. These findings not only deepen the understanding of damage mechanisms under low-velocity impact but also provide a valuable foundation for practical engineering applications.

### 2.2. Fatigue and Fracture

Fatigue and fracture behavior are of fundamental importance in the mechanical design of composite materials, as they directly govern the durability, reliability, and safety of engineering structures.

In the paper entitled “Fatigue Behavior of Cord-Rubber Composite Materials under Different Loading Conditions”, Torggler et al. [14] employed a combination of numerical simulations and experimental methods to evaluate the fatigue life of cord–rubber composites. Their results demonstrated that a higher strain ratio leads to an extended service life, as evidenced by a direct comparison between different loading conditions. This finding provides useful insight into the optimization of fatigue performance in such materials.

Liu et al. [15], in “Fracture Performance Study of Carbon-Fiber-Reinforced Resin Matrix Composite Winding Layers under UV Aging Effect”, investigated the influence of ultraviolet (UV) irradiation duration and temperature on the Mode I, Mode II, and mixed-mode fracture toughness of composite laminates. Their study revealed that Mode I fracture toughness increases with prolonged UV exposure, whereas Mode II fracture toughness exhibits a significant reduction. These contrasting trends highlight the complex effects of environmental aging on fracture behavior.

Collectively, these contributions offer important insights into the fatigue and fracture mechanisms of composite materials and are of particular relevance to applications in the automotive industry and hydrogen storage systems.

### 2.3. Bonding Strength

The strength of composite materials, which is strongly influenced by manufacturing processes, remains a central concern in the design of engineering structures. In recent years, additive manufacturing, particularly three-dimensional (3D) printing, has significantly transformed the industrial landscape by enabling the fabrication of complex and highly customized geometries. However, its application in load-bearing structures is still constrained by limitations in interfacial bonding strength.

In this context, Hümbert et al. [16], in their paper entitled “Modelling of Bond Formation during Overprinting of PEEK Laminates”, investigated the applicability of healing models to the overprinting process of thermoplastic composite laminates. Their study demonstrated that the thermal history at the interface, especially the deposition conditions of the initial layer, plays a decisive role in achieving effective in situ bonding. These findings provide fundamental insights into the mechanisms governing interfacial strength and establish essential guidelines for future process optimization and component development. Consequently, this work contributes to advancing the technological maturity of overprinting in composite manufacturing.

### 2.4. Lightning Strike

Lightning strike events pose significant challenges to the structural integrity and functional performance of composite materials, particularly in aerospace, wind turbine blades, and infrastructure applications.

Wang et al. [17], in their review article entitled “Challenges and Future Recommendations for Lightning Strike Damage Assessments of Composites: Laboratory Testing and Predictive Modeling”, comprehensively elucidate the role of simulated lightning strike experiments in providing critical inputs for damage modeling, as well as high-quality experimental data for model validation. The review offers a systematic and holistic perspective on the current state of the field, identifying existing capabilities, key limitations, and unresolved challenges in both experimental testing and predictive modeling.

Furthermore, the authors present a series of forward-looking recommendations aimed at enabling robust and high-fidelity prediction of lightning strike damage in composite materials. The insights derived from this work are expected to drive further advancements in enhancing the safety, reliability, and durability of composite structures subjected to lightning strike conditions.

### 2.5. Others

Beyond mechanical behavior, other functional properties of composite materials and structures—such as magnetic, optical, radiation tolerance, thermal, and electrical characteristics—are also of considerable interest for a wide range of advanced engineering applications.

Ebrahimi et al. [18], in their comprehensive review entitled “Enhanced Multifaceted Properties of Nanoscale Metallic Multilayer Composites”, highlight the remarkable performance enhancements achieved through precise control of layer thickness and interfacial characteristics in nanoscale metallic multilayer composites. The authors systematically compile relevant experimental findings alongside existing and emerging applications, providing detailed discussions on fundamental material attributes, synthesis techniques, and commonly employed characterization methods.

In addition, the authors critically examine current challenges and outline future research directions in the field of nanostructured composites. Overall, this work serves as a valuable resource for researchers and engineers seeking to fully exploit the multifunctional potential of nanoscale metallic multilayer composites in advanced technological applications.

## 3. Outlook

This Special Issue of *Materials* has received strong support from the research community, culminating in the publication of eight high-quality, rigorously peer-reviewed articles. As Guest Editor, I sincerely appreciate the valuable contributions of all authors to this collection.

With the increasing demand for high-performance materials and the growing need for the integration of structural and functional capabilities [19], the design of advanced composite materials and structures is becoming progressively more complex and challenging. Consequently, there is an urgent need for innovative methodologies and emerging technologies to overcome existing bottlenecks in manufacturing, design, and performance analysis. In this context, the advent of artificial intelligence (AI) is driving a transformative shift toward data-driven approaches, which are rapidly permeating research in composite materials and structures [20,21,22,23]. These advancements hold significant promise for accelerating discovery, improving predictive capabilities, and enabling more efficient design strategies. Looking ahead, such developments may ultimately lead to the identification of the fundamental performance limits of advanced composite materials and structures, thereby unlocking new frontiers in engineering applications.
